# Peripheral ulcerative keratitis, nodular episcleritis, and pulmonary nodules as the initial signs of rheumatic arthritis: A Case Report

**DOI:** 10.3389/fimmu.2022.1048889

**Published:** 2022-11-10

**Authors:** Jingting Wang, Qin Zhang, Weiyun Shi, Yilin Pang, Suxia Li

**Affiliations:** ^1^ Eye Institute of Shandong First Medical University, Eye Hospital of Shandong First Medical University, Shandong Eye Hospital, State Key Laboratory Cultivation Base, Shandong Provincial Key Laboratory of Ophthalmology, School of Ophthalmology, Shandong First Medical University, Jinan, China; ^2^ Department of Ophthalmology, Peking University People’s Hospital, Eye Diseases, and Optometry Institute, Beijing Key Laboratory of Diagnosis and Therapy of Retinal and Choroid Diseases, College of Optometry, Peking University Health Science Center, Beijing, China

**Keywords:** peripheral ulcerative keratitis, nodular episcleritis, rheumatoid vasculitis, rheumatoid arthritis, pulmonary nodules, infliximab

## Abstract

**Background:**

Rheumatoid vasculitis (RV) is a rare but potentially devastating complication of rheumatoid arthritis (RA). It typically occurs in patients with extra-articular manifestations. Here we reported a case of PUK with nodular episcleritis and pulmonary nodules that occurred in the same patient without joint involvement.

**Case presentation:**

A 43-year-old Chinese woman, exhibited a partial crescent-shaped marginal corneal ulcer in the right eye at admission and the ulcer developed rapidly into nearly 360-degree ulcers in both eyes within one week. Nodular episcleritis was observed in the right eye. Conjunctival biopsy revealed vasculitis. Her rheumatoid factor (RF) and anti-cyclic citrullinated protein antibody were positive, while anti-neutrophilic cytoplasmic antibody (c-ANCA) and anti-protease 3 were negative. Pulmonary nodules were found, without joint involvement. The ocular condition did not relieve under the topical and systemic use of corticosteroids, or under other immunosuppressive agents until the infliximab therapy. PUK recurrence was observed after the discontinuation of infliximab.

**Conclusions:**

Rapidly deteriorated PUK with nodular episcleritis and pulmonary nodules occurred in the same patient is a special case of RA without joint involvement. This case reinforces the concept that RV may be the initial sign of RA. Infliximab can be used to prevent further progress of RA-related PUK in some refractory cases.

## Background

Rheumatoid arthritis (RA) is a chronic inflammatory disease characterized by joint swelling, joint tenderness, and destruction of synovial joints, but is sometimes complicated by extra-articular conditions, including subcutaneous nodules, lung involvement, pericarditis, peripheral neuropathy, vasculitis, and ocular disease (1). Rheumatoid vasculitis (RV) is a rare, extra-articular manifestation in patients with long-standing seropositive RA. As a general rule, RA is its leading cause, ahead of systemic vasculitis. The mean duration between the diagnosis of RA and the onset of vasculitis is 10–14 years (2). Here we describe a rare case of rapidly deteriorated bilateral peripheral ulcerative keratitis (PUK) with nodular episcleritis and pulmonary nodules that occurred in the same patient as the initial signs of RA without joint involvement.

## Case presentation

**Figure 1:**
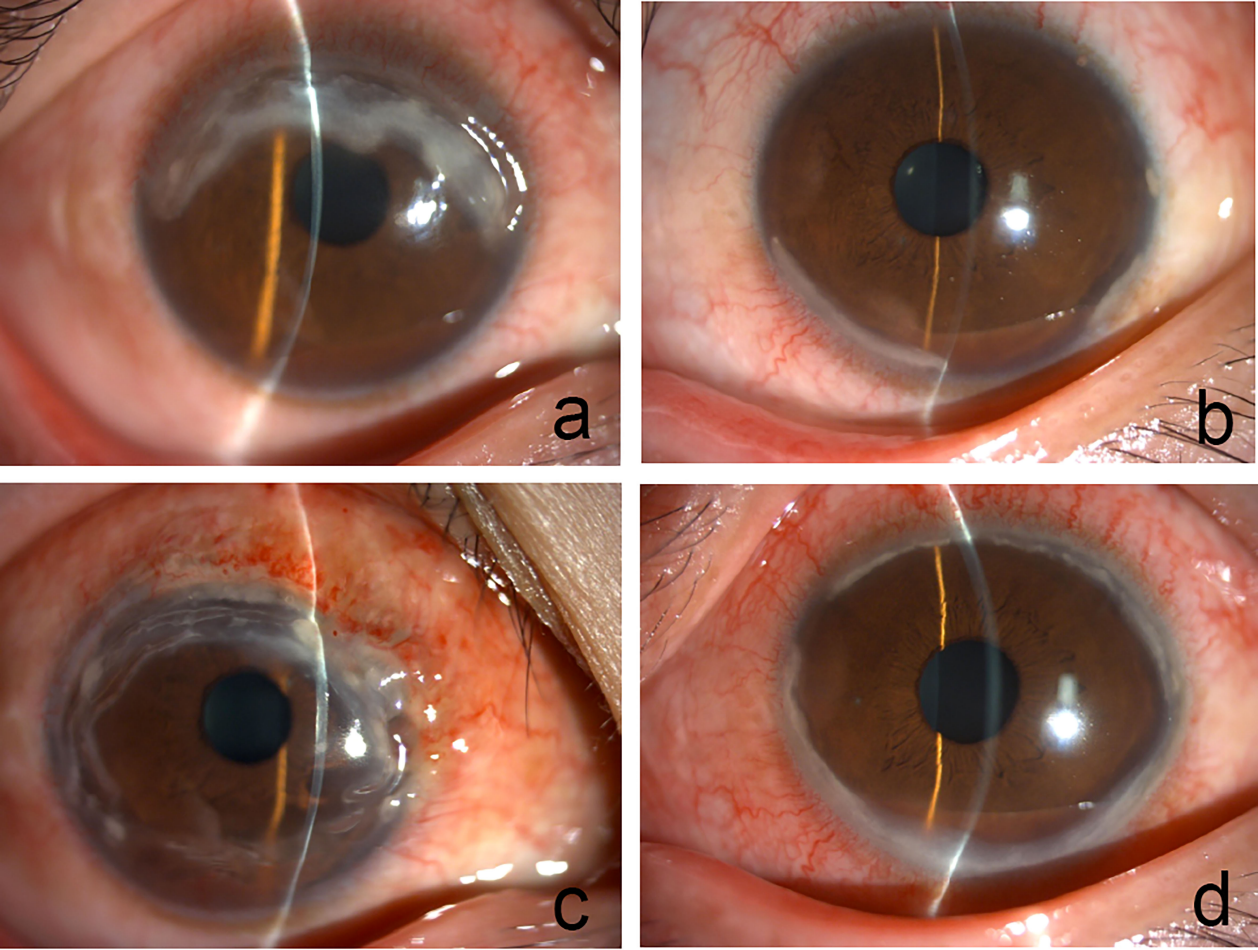
**(A)** Slit lamp reveals corneal ulcer was noted at 9 to 3 o’clock position in the right eye. **(B)** Conjunctival congestion and peripheral ulcerative keratitis in the left eye. **(C)** Nodular episcleritis appeared in the right eye. **(D)** 360-degree ring ulcer in the left eye.

In 2018, a 43-year-old Chinese woman complained about a 14-day history of progressing pain and redness in her right eye. She had no ocular disease and medical history. There was no evidence of inflammatory arthritis or other symptoms of connective tissue disease. The best-corrected visual acuity was 20/40 in the right eye and 20/20 in the left eye. A crescent-shaped marginal corneal ulcer was noted from the 9 to 3 o’clock position on the right eye (**Figure 1A**). The cornea of the left eye was normal. There was no other evidence of posterior segment involvement in both eyes. Bacterial and fungal cultures were negative in corneal scrapings. The results of serum immunological examination were as follows: RF, 104 IU/ml (normal range, 0-20 IU/mL); anti-CCP antibody, 65.29 U/mL (normal range, 0-17 U/mL); anti-nuclear antibody (ANA), 1:100 (normal range, 1:100); anti-mutated citrullinated vimentin (anti-MCV), 303.6 U/mL (normal range, 0-20 U/ml). Antibodies against the glomerular basement membrane were negative. In addition, c-ANCA, anti-protease 3, and HLA-B27 were negative. Related laboratory results and minimum/maximum values are in **Supplementary Table 1**. The primary diagnosis was PUK in the right eye and its association with autoimmune disease was highly suspected. Treatment with topical tacrolimus and tobramycin dexamethasone eye drops was initiated but was found to be ineffective after a three-day observation. Slit-lamp examination showed a newly emerged conjunctival congestion and peripheral ulcerative keratitis was observed in the left eye (**Figure 1B**). In addition to the eye drops of topical tacrolimus and tobramycin dexamethasone in both eyes, intravenous cyclophosphamide (200 mg, once every 2 weeks) and methylprednisolone (40 mg/day) were also given immediately.

**Figure 2:**
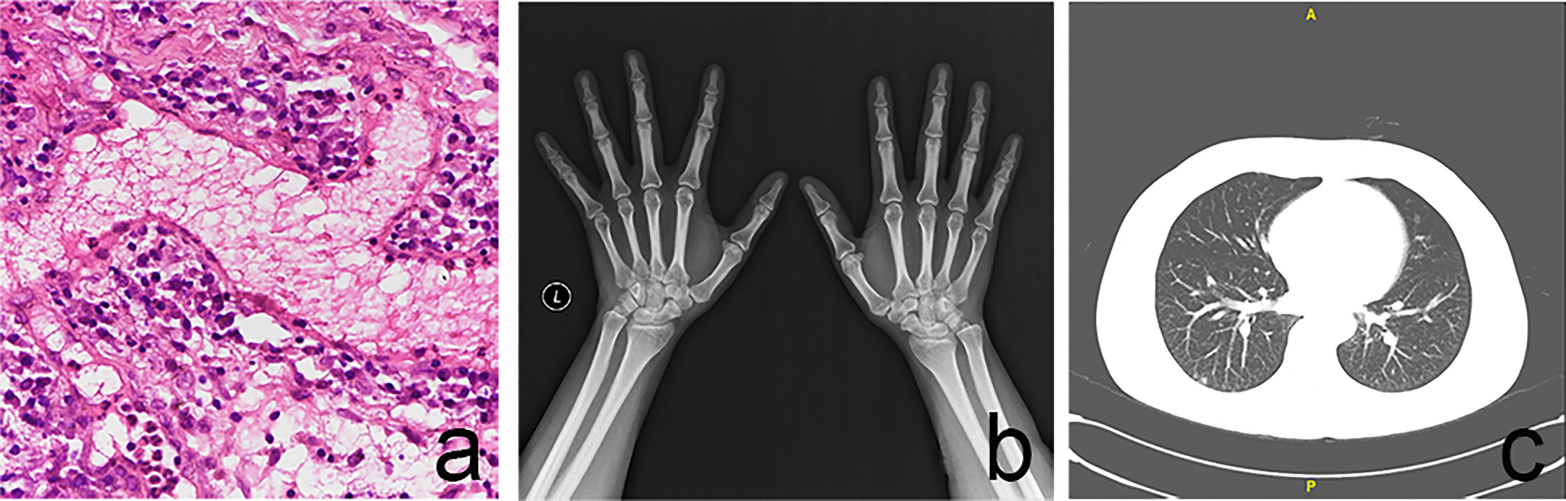
**(A)** The conjunctival biopsy of the right eye revealed vascular proliferation, telangiectasis, perivascular lymphocytes and plasma cells infiltration. **(B)** Hands and wrists X-rays with no evidence of bony erosion. **(C)** Chest CT showed multiple nodules in the inferior lobe of both lungs.

**Figure 3:**
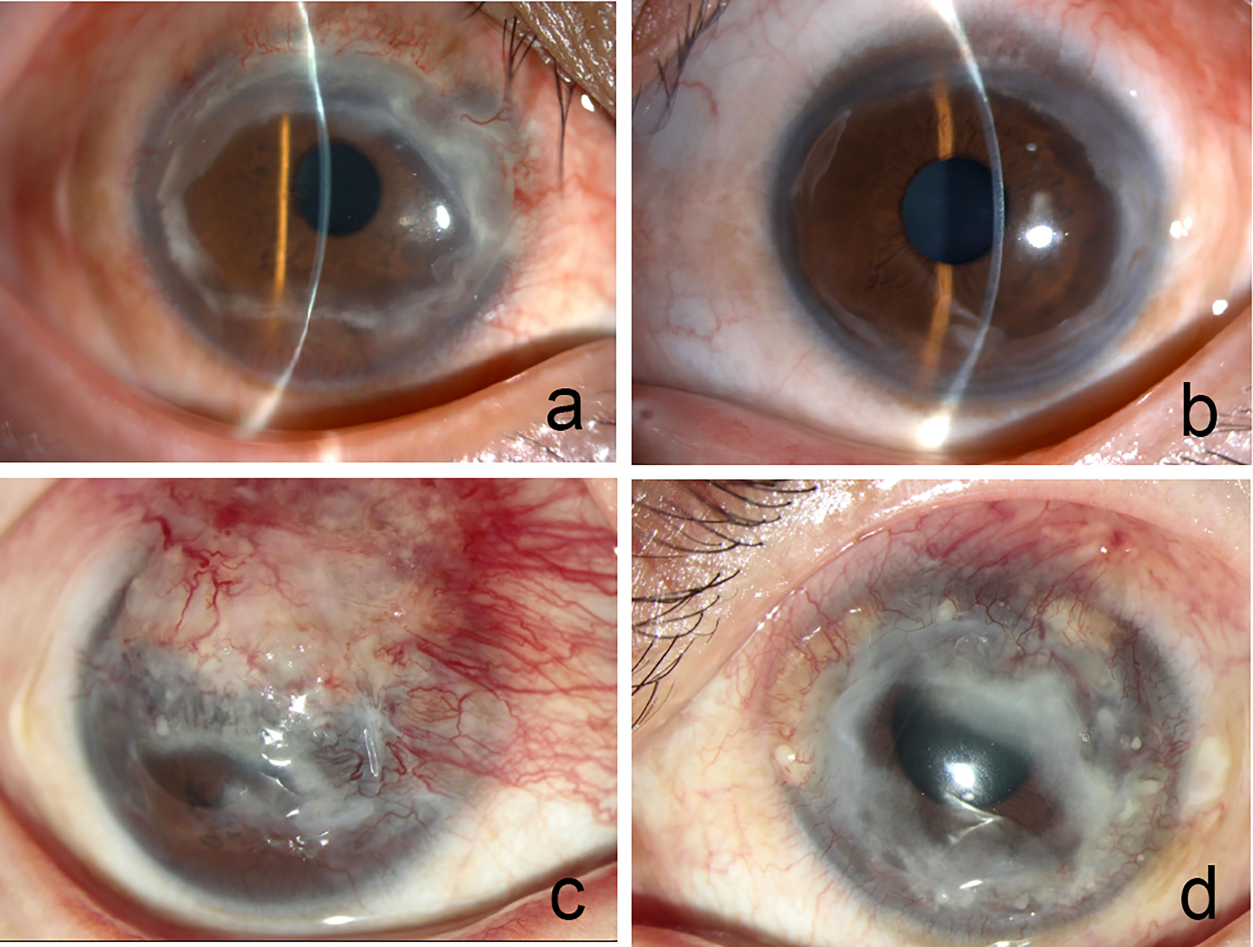
Corneal ulcers almost healed up in both eyes (**A** right eye, **B** left eye). the superior two-thirds of the cornea was covered by neovascularization and necrotic tissue in the right eye (**C** right eye). Corneal ulceration was observed throughout the cornea except for the central cornea (**D** left eye).

However, the corneal ulcers were not well controlled and rapidly developed into nearly 360-degree ulcers in both eyes within one week. Nodular episcleritis appeared in the right eye (**Figures 1C, D**). The conjunctival biopsy of the right eye revealed vascular proliferation, telangiectasis, perivascular lymphocytes and plasma cells infiltration (**Figure 2A**). At the same time, the patient received a further systemic examination. No obvious abnormality was found in joint X-rays on both hands and wrists (**Figure 2B**). Chest CT showed multiple nodules in the inferior lobes of both lungs (**Figure 2C**). The laboratory results showed no apparent abnormality in antiphospholipid antibody, interferon detection, mixed lymphocyte culture, tuberculosis testing, thyroid function, renal function, and an ultrasonic cardiogram. Infliximab (3 mg/kg for three starter doses at 0, 2, and 6 weeks respectively, and the same dose every 8 weeks after the three starter doses) and an increased dose of 60 mg/day oral prednisolone following pulsed methylprednisolone (1,000 mg/day for 3 consecutive days per week) twice were administered. The oral prednisolone was continued at a stable dose for four weeks and then gradually tapered by 5 mg every ten days. At the 2-month follow-up visit, corneal ulcers almost healed up in both eyes (**Figures 3A, B**).

**Figure 4:**
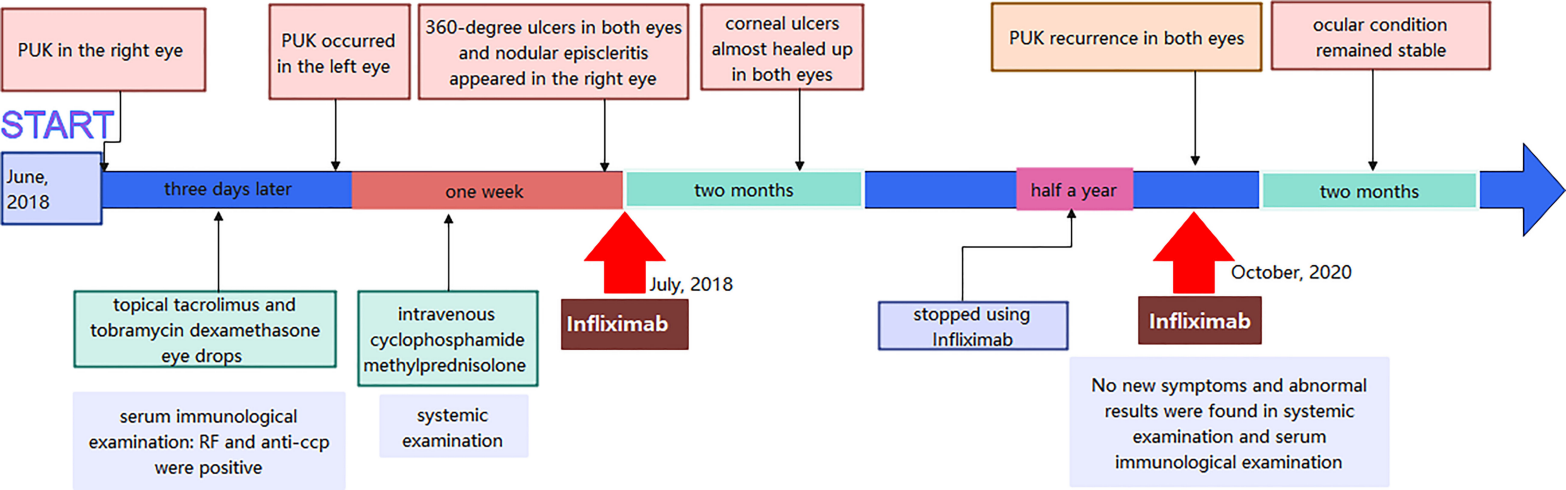
Timeline of the case report.

However, PUK recurrence in both eyes was observed two years later, half a year after she stopped using Infliximab due to the impact of the COVID-19 pandemic in 2019. Slit-lamp examination showed that the superior two-thirds of the cornea was covered by neovascularization and necrotic tissue in the right eye, and corneal ulceration was observed throughout the cornea except for the central cornea in the left eye (**Figures 3C, D**). No new symptoms and abnormal results were found in systemic examination and serum immunological examination, respectively. Infliximab (5 mg/kg every 4 weeks) and an increased dose of 60 mg/day of oral prednisolone were administered again besides the topical eye drops. At the 2-month follow-up visit, the patient’s ocular condition remained stable. Her visual acuity was hand movements in the right eye and 20/400 in the left eye. She was suffered from the loss of vision and under great pressure of huge medical fees. **Figure 4** was showcasing a timeline with relevant data from the episode of care.

## Discussion and conclusions

RV is the most serious extra-articular complication of RA, and typically occurs in patients with long-standing, erosive, deforming RA. Involvement of ocular, lung, kidney and central nervous system is much rarer but can be organ-threatening or life-threatening in RV (2). In this case, the patient did not have joint involvement, which cannot be classified as definite RA by now according to the 2010 ACR-EULAR diagnostic criteria (3). Indeed, there are several lines of evidence supporting the diagnosis of RA. First, the positive anti-CCP and RF suggested the diagnosis of RA. Various studies have shown that the specificity of anti-CCP in combination with RF for RA was 98~100% (4, 5). Second, the conjunctival biopsy from the right eye of this patient was compatible with an autoimmune-mediated vasculitis. Last, PUK, nodular episcleritis, and pulmonary nodules that occurred in the same patient can be considered as manifestations of RV. Previous case reports separately reported lung disease or PUK as the first manifestation of RV may predate the articular manifestations of RA (6, 7). Nodular episcleritis is an uncommon condition in RA, with sectorial vasodilation of the deep episcleral vessels, forming a localized inflammatory nodular aspect (8). Above contents suggested the onset of RV may be earlier than RA.

It was difficult to distinguish the possibility that this case represented an atypical presentation of GPA because PUK and pulmonary nodules can be found in both GPA and RA patients. A meta-analysis revealed that the risk of GPA and RA might share similar pathogenic mechanisms (9). A previous study reported that there might be some GPA patients overlapping RA (10). Early identification of the GPA was based on the combination of histopathologic findings in a biopsy, positive ANCAs, and the presence of pulmonary, upper respiratory, and/or renal manifestations. In this case, the patient had a high-titer RA-specific autoantibody (RF and anti-CCP), and ANCA as a highly specific indicator for diagnosis of GPA was negative. The overall clinical scenario favored RA as the best unifying diagnosis.

RA-related PUK is a medical emergency, not only because it can cause perforations, but also because of the underlying systemic vasculitis which is lethal frequently. Treatments of PUK aim to reduce inflammation, promote epithelial healing, and minimize stromal loss, which includes topical treatment, surgical treatment, and systemic treatment. In our case, the use of intravenous cyclophosphamide in combination with corticosteroids was ineffective, but infliximab therapy obtained a good effect. Infliximab, a chimeric monoclonal antibody specific for the key proinflammatory cytokine tumor necrosis factor-alpha (TNFα), was reported effective and safe for the treatment of RA-related PUK (11). Currently, antitumors necrosis factor (anti-TNF) therapy has been proved as an efficacious therapeutic strategy in RA (12, 13). Besides, PUK recurrence was observed after the discontinuation of infliximab. This suggests the necessity of maintenance therapy.

In conclusion, we strongly suspect that rapidly deteriorated PUK with nodular episcleritis and pulmonary nodules occurred in the same patient is a special case of RA without joint involvement. This case reinforces the concept that RV may be the initial sign of RA. Infliximab can be used to prevent further progress of RA-related PUK in some refractory cases.

## Data availability statement

The raw data supporting the conclusions of this article will be made available by the authors, without undue reservation.

## Ethics statement

The studies involving human participants were reviewed and approved by ethics committee of the Eye hospital of Shandong First Medical University. The patients/participants provided their written informed consent to participate in this study. Written informed consent was obtained from the individual(s) for the publication of any potentially identifiable images or data included in this article.

## Author contributions

JW and QZ Investigated and drafted this manuscript. SL performed treatment for the patient and edited a draft of the manuscript as the corresponding author. WS and YP supervised and critically reviewed this article. All authors contributed to the article and approved the submitted version.

## Acknowledgments

We greatly appreciate that Peking University People’s Hospital gave systemic examination and treatment.

## Conflict of interest

The authors declare that the research was conducted in the absence of any commercial or financial relationships that could be construed as a potential conflict of interest.

## Publisher’s note

All claims expressed in this article are solely those of the authors and do not necessarily represent those of their affiliated organizations, or those of the publisher, the editors and the reviewers. Any product that may be evaluated in this article, or claim that may be made by its manufacturer, is not guaranteed or endorsed by the publisher.

## Abbreviations

PUK, Peripheral ulcerative keratitis; RA, rheumatoid arthritis; RF, rheumatoid factor; Anti-CCP, anti-cyclic citrullinated protein antibody; c-ANCA, anti-neutrophilic cytoplasmic antibody; GPA, granulomatosis with polyangiitis; ANA, anti-nuclear antibody; anti-MCV, anti-mutated citrullinated vimentin; TNFα, tumor necrosis factor-alpha.
