# Supplementary material for: Peripheral ulcerative keratitis, nodular episcleritis, and pulmonary nodules as the initial signs of rheumatic arthritis: A Case Report
Source: Front Immunol. 2022 Nov 10;13:1048889. doi: 10.3389/fimmu.2022.1048889 (PMC9686418; doi:10.3389/fimmu.2022.1048889)
Supplement: Supplementary file 1 [file Table_1.docx]

**Supplementary Tables:**

**Supplementary Table 1: Overview of laboratory parameters and autoimmune serologies**

| **Parameter (Unit)** | **value** | **Normal range** |
| --- | --- | --- |
| RF (IU/ml) | 104 | 0 - 20 |
| Anti-CCP (U/ml) | 65.29 | 0 - 17 |
| Anti-MCV (U/ml) | 303.6 | 0 - 20 |
| ANA | 1:100 | < 1:100 |
| SSA (RU/ml) | 3.69 | 0 - 20 |
| SSB (RU/ml) | <2.00 | 0 - 20 |
| dsDNA (U/ml) | <10 | 0 - 100 |
| MPO (RU/ml) | <2 | 0 - 20 |
| Proteinase 3 ANCA (RU/ml) | <2 | 0 - 20 |
| Myeloperoxidase ANCA (U/ml) | <5 | 0 - 5 |
| Perinuclear ANCA | <1:10 | < 1:10 |
| Cytoplasmic ANCA | <1:10 | < 1:10 |
| Antiglomerular basement membrane antibody (RU/ml) | 1.1 | 0 - 20 |
| Erythrocyte sedimentation rate (mm/h) | 10 | 0 - 20 |
| CRP (mg/L) | < 5.0 | 0 -10.0 |
| HLA B27 | neg. | neg. |
| ASO (KU/L) | 95.1 | 0-200 |
| IgA (g/L) | 3.67 | 0.7-4.0 |
| IgG (g/L) | 16.7 | 7 -16 |
| IgM (g/L) | 1.09 | 0.4 - 2.30 |
| IgE (IU/ml) | 61.30 | 0 - 100 |
| C3 (g/L) | 1.1 | 0.8 -1.8 |
| C4 (g/L) | 0.16 | 0.1 - 0 |
| ACL – IgA (CU) | < 1.40 | 0 - 1.5 |
| ACL - IgG (CU) | < 2.60 | 0 - 17 |
| ACL - IgM (CU) | 2.1 | 0 -10 |
| β 2GP1-IgA (CU) | < 4.00 | 0 - 4 |
| β 2GP1-IgG (CU) | < 6.40 | 0 - 6.5 |
| β 2GP1-IgM (CU) | < 1.10 | 0 – 4.5 |
| Troponin I (ng/ml) | < 0.002 | 0 – 0.03 |
| T-SPOT.TB | negative | negative |
| β-D-glucan test (pg/ml) | 36.68 | < 60 |
